# Portable Head-Mounted System for Mobile Forearm Tracking

**DOI:** 10.3390/s24072227

**Published:** 2024-03-30

**Authors:** Matteo Polsinelli, Alessandro Di Matteo, Daniele Lozzi, Enrico Mattei, Filippo Mignosi, Lorenzo Nazzicone, Vincenzo Stornelli, Giuseppe Placidi

**Affiliations:** 1 DISA-MIS, University of Salerno, 84084 Fisciano, Italy; 2 A2VI-Lab, DISIM, University of L’Aquila, 67100 L’Aquila, Italy; alessandro.dimatteo1@graduate.univaq.it (A.D.M.); daniele.lozzi@graduate.univaq.it (D.L.); enrico.mattei@graduate.univaq.it (E.M.); filippo.mignosi@univaq.it (F.M.); 3 A2VI-Lab, DIIIE, University of L’Aquila, 67100 L’Aquila, Italy; lorenzo.nazzicone@student.univaq.it (L.N.); vincenzo.stornelli@univaq.it (V.S.); 4 A2VI-Lab, c/o Department of MESVA, University of L’Aquila, 67100 L’Aquila, Italy; giuseppe.placidi@univaq.it

**Keywords:** hand tracking, leap motion controller, Raspberry Pi, portable device, wearable device

## Abstract

Computer vision (CV)-based systems using cameras and recognition algorithms offer touchless, cost-effective, precise, and versatile hand tracking. These systems allow unrestricted, fluid, and natural movements without the constraints of wearable devices, gaining popularity in human–system interaction, virtual reality, and medical procedures. However, traditional CV-based systems, relying on stationary cameras, are not compatible with mobile applications and demand substantial computing power. To address these limitations, we propose a portable hand-tracking system utilizing the Leap Motion Controller 2 (LMC) mounted on the head and controlled by a single-board computer (SBC) powered by a compact power bank. The proposed system enhances portability, enabling users to interact freely with their surroundings. We present the system’s design and conduct experimental tests to evaluate its robustness under variable lighting conditions, power consumption, CPU usage, temperature, and frame rate. This portable hand-tracking solution, which has minimal weight and runs independently of external power, proves suitable for mobile applications in daily life.

## 1. Introduction

Wearable devices (WDs) use sensors installed directly on the hand and fingers to perform hand tracking and gesture recognition, making them haptic and precise. However, WDs are expensive, and their major limitation is their dependence on hand size and shape, which can greatly limit movements and/or require continuous adaptations.

In contrast, by using cameras surrounding the scene and recognition algorithms, computer vision (CV) is very useful for developing touchless hand-tracking and gesture recognition systems [1,2,3] that are low-cost, precise, and have high versatility in recognizing every hand size and silhouette without the necessity of re-implementation or adaptation. The primary benefit of CV-based systems lies in their ability to operate without physical contact, allowing for unrestricted, fluid, and natural movements. Moreover, the hand is not constrained by any wearable device, enabling it to naturally grasp a wide range of daily-use tools.

This is why CV-based systems are gaining popularity in several fields, like human-system interaction, virtual reality environments, and even remote operations for medical procedures and rehabilitation [4].

Usually, CV-based hand tracking uses cameras placed in a fixed position, for example, on a desk, and a computer to run CV-based strategies for tracking. However, this configuration limits daily interactions and makes these technologies incompatible with mobile or ubiquitous applications [5]. Indeed, CV-based technologies usually require remarkable computing capabilities, especially when real-time applications with a minimum number of frames are required. As a consequence, the tracking systems cannot be transported easily or with minimal hindrance.

In systems featuring stationary cameras, users are also obliged to carry out their tasks within a predetermined field of view, restricting their activities to a specific location. A more effective solution would be to change the position of the hand-tracking sensor to be consistent with the user’s field of view, for example, placing the sensor on the user’s head.

This requirement is even more evident when virtual reality (VR) or augmented reality (AR) headsets are used. These systems are typically composed of a head-mounted display (HMD) and a physical controller or a dedicated device for hand tracking. Enabling controller-free interaction with virtual environments would allow one to grasp real-world objects, making the interaction natural and immersive [6], which is recommended, especially in AR applications.

In the last few years, several manufacturers have integrated sensors (RGB, IR, depth, or a combination of them) directly into their headsets, using CV-based strategies for hand tracking [7].

One of these sensors, already market-available and widely used, is the Leap Motion Controller (LMC) [8,9,10].

The LMC offers precise hand tracking, a high frame rate, and adequate latency [11,12] at an affordable price and compact dimensions [13].

Moreover, the LMC’s software, in its recent release, has implemented a head-mounted option that enables tracking the hand from the back instead of the palm. Therefore, both the hardware and software features make the LMC particularly suitable for grasping applications.

Head-mounted hand tracking is an effective design choice because the user’s hands are free to interact with the world without worrying about the position of the hand-tracking sensor, which consistently captures the user’s field of view [14]. Nevertheless, a limitation remains: the presence of a computer for hand-tracking operations strongly limits the portability of the system.

With the recent advancements in single-board computers (SBC), powerful, compact, and portable computing devices have become available. These devices are capable of managing LMC sensors at sufficient frame rates [15].

**Figure 1:**
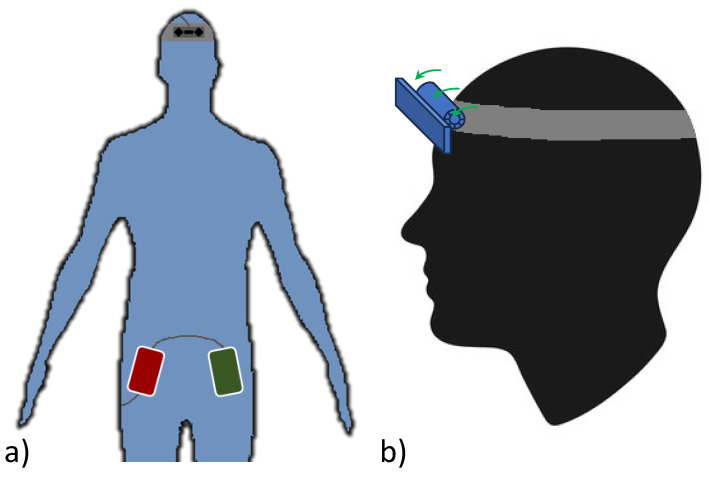
System embodiment: (**a**) pocket location of the SBC and power bank with respect to the head-mounted LMC; (**b**) details of the LMC support that enables changing the vertical orientation of the sensor.

In this work, we present a portable hand-tracking system based on an LMC (version 2 released in 2023) controlled by an SBC powered by a power bank. Figure 1a illustrates an embodiment of the proposed system: the LMC is mounted on the head and connected to the SBC powered by a power bank. Both the SBC and power bank are compact and can be carried in a pocket. Head-mounted support has been adapted to accommodate the LMC, allowing for the adjustment of the orientation of the sensor (as shown in Figure 1b) according to the user’s requirements.

In this embodiment, the proposed system can be adapted for several applications, leaving both hands completely free. Moreover, the system is independent of an external power supply and its weight is minimal, making it suitable for outdoor applications.

The remainder of this paper is organized as follows. Section 2 reports the related works; Section 3 describes the proposed system in detail; Section 4 presents a use case and some experimental tests, assessing the robustness of the proposed device under variable lighting conditions, as well as its power consumption, CPU usage, temperature, and frame rate; and finally, Section 5 presents the conclusions and outlines future work.

## 2. Related Works

One of the first works to use a head-mounted sensor for 2D hand-gesture recognition was [14], where only a single RGB camera was used to minimize the weight.

In [16], a head-mounted gaze-directed camera was used for 3D hand-tracking of everyday manipulation tasks. However, the system needed a powerful computational machine to achieve tracking at 12 frames per second (fps).

In [17], two RGB head-mounted synchronized cameras were proposed (the two cameras were calibrated offline) for 3D hand-gesture recognition, similar to [16]. The captured videos, with frames sized at 320 × 240, were processed at about 30 fps using a dedicated personal computer. The RGB system was lightweight (65 g), but its bottleneck was the personal computer, which was heavy and bulky.

In 2013, the first version of the LMC was presented for 3D hand tracking. It was a revolutionary device, being very lightweight (only 32 g), compact (80 × 30 × 11.30 mm), fast (between 40 fps to 120 fps, depending on the managing hardware), and precise (about 1 mm) [18,19]. In the beginning, the LMC was designed to be placed and used on a desk and connected to a personal computer, and several works appeared using it in disparate applications [8,20,21,22,23,24].

However, the sensor’s features made it very suitable for head-mounted hand tracking, and in 2014, the software of the LMC was updated to support VR tracking mode, designed to provide hand tracking while the device was mounted on virtual reality headsets [25].

In the following years, several works were presented using the LMC in a head-mounted assembly, also in combination with VR/AR headsets [6,26,27,28,29,30,31], confirming the sensor’s suitability for this type of task.

Nowadays, several VR/AR headsets have built-in hand-tracking capabilities, like Microsoft Hololens 2 [32,33,34] and Meta Quest [35,36].

However, in general, VR/AR headsets either continue to be connected to a personal computer (which limits their usable space) or have expensive built-in CPUs that mostly hinder access to raw data (such as Meta Quest [37]).

Recently, in [38], the authors proposed a system based on an LMC connected to a mini PC powered by a very powerful power bank. The LMC is head-mounted, whereas the mini PC and power bank are placed inside a backpack that the user has to wear. Indeed, this design allows for complete mobility by the user but is uncomfortable. As the authors pointed out, the LMC software requires an X_86 machine, limiting the possibility of using a lightweight SBC. However, in [15], the authors demonstrated that it is possible to run the LMC software on an SBC equipped with an ARM CPU architecture through virtualization, reaching almost 30 fps. The SBC used was a Raspberry Pi 4 Model B [39] (Raspberry Pi Ltd., Cambridge, UK), which we here abbreviate as RSP.

**Figure 2:**
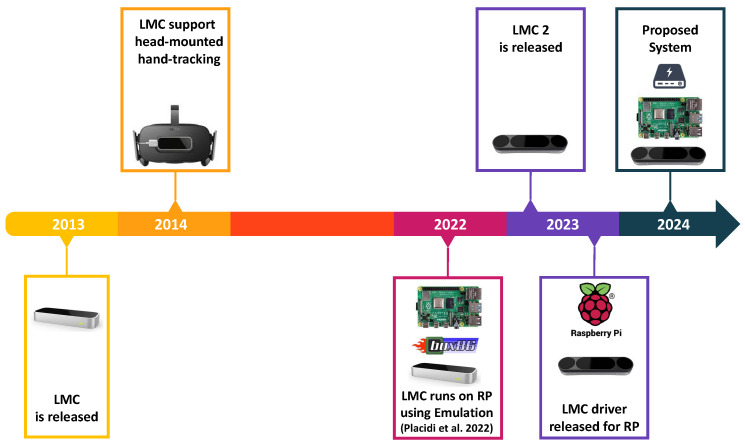
Timeline of the LMC: The sensor was first designed to be driven by a personal computer. In recent embodiments over the last two years, it has started to be driven by an SBC (Placidi et al. 2022 [15]). Further, in its latest release, the ROI was increased and back-hand tracking was enabled, allowing for complete head-mounted embodiments.

Finally, in December 2023, the LMC manufacturer released a new version that supports the RSP [40,41]. Figure 2 depicts the timeline of the LMC.

The design and development of a portable forearm-tracking system requires satisfying several critical requirements: reduced costs and energy consumption, increased battery life, decreased temperature, reduced CPU usage, and maximized frame rate, among others. Without meeting these requirements, a forearm-tracking system would be incomplete. The system we propose herein satisfies the requirements for complete forearm tracking with an extensible ROI. Moreover, we have also kept the cost of the system low without compromising precision.

## 3. System Design

The LMC is a compact device designed for precise hand and finger tracking in three-dimensional space. Measuring just 84 mm × 20 mm × 12 mm and weighing only 29 g, it offers portability and ease of use. With low power consumption requirements of 5 volts and 500 milliamps, it efficiently integrates into various setups without compromising performance [42]. The controller uses a combination of infrared sensors and cameras to track the movements of hands and fingers with high accuracy [18]. The drivers for the LMC are compatible with a wide range of desktop CPUs, operating systems, and VR/AR headsets. Recently, they have also been made available for the RSP, resulting in minimized energy consumption and optimized performance for the LMC itself.

The RSP used is equipped with a Broadcom BCM2711 CPU (Broadcom Inc., North San Jose, CA, USA), Quad-core Cortex-A72 (ARM v8) 64-bit SoC at 1.8 GHz, and 4 GB of RAM, with the RSP enclosed in its case. This is a powerful and low-cost RSP with low dimensions and weight (88 mm × 58 mm × 19.5 mm and 46 g) and low energy consumption (max 15 W). As a result, the RSP stands out among the SOCs that best meet the requirements of the proposed system.

Given that the LMC now supports the RSP, the natural synergy between them makes their combination an obvious choice. Together, they provide precise hand-tracking capabilities, enhancing the overall functionality and usability of the system. Moreover, the proposed system is conveniently powered by an external portable power supply, ensuring flexibility and ease of use in various settings.

In fact, through a USB-C port, the RSP is connected to a power bank (with dimensions of 105 mm × 68 mm × 20 mm, weight of 190 g, and capacity of 10,000 mAh) capable of providing a maximum power of 20 W. The RSP is connected to the LMC through a USB 3.0 port.

**Figure 3:**
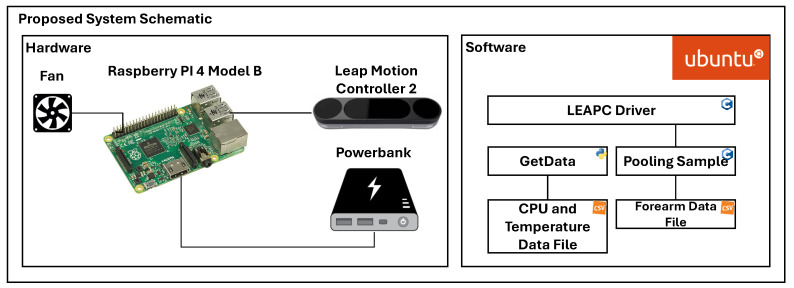
System architecture.

**Figure 4:**
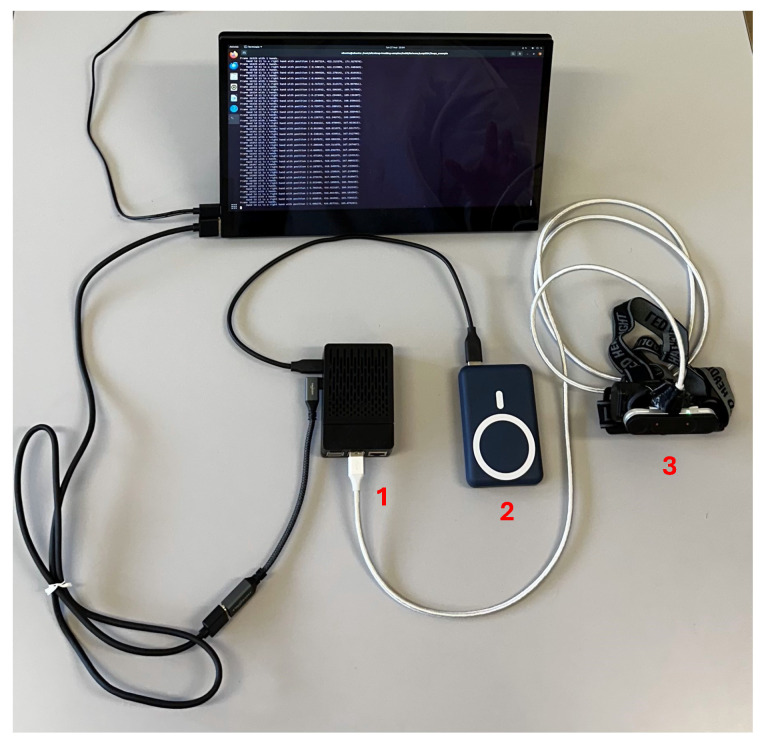
System embodiment: (1) the RSP enclosed in its case, (2) the power bank, (3) the LMC inside the head-mounted support. The presence of the monitor, not used in the final embodiment, is necessary during system calibration to set the values of the parameters.

The design of the proposed system is shown in Figure 3. The RSP is placed into a protective case (not shown in Figure 3 but indicated as 1 in Figure 4) that prevents it from being exposed to dust, humidity, and impacts that could damage the board. The RSP is equipped with passive heat dissipation (heatsinks) and a fan for active dissipation to avoid overheating due to the high-intensity operations required for tracking, both hosted in the protective case.

The RSP runs on the Ubuntu 20.04 operating system optimized for ARM architectures. The official LMC software for the RSP was installed, and the LeapC API was used for the development of the scripts for data acquisition.

The “Pooling Sample” script, provided with the LMC software, was modified to enable data saving. The initial modification was to set the acquisition mode to head-mounted, running a specific function defined in the LeapC API. After the conclusion of this phase, data frame streaming was initialized. For each frame, data regarding the frame ID, the frame rate, the hand IDs, and all the coordinates of the joints of each hand are extracted and saved in a CSV file. Figure 3 also reports the “GetData” script, developed in Python 3.9, which, although not part of the final system embodiment, is used to collect data on CPU usage and temperature behavior.

**Figure 5:**
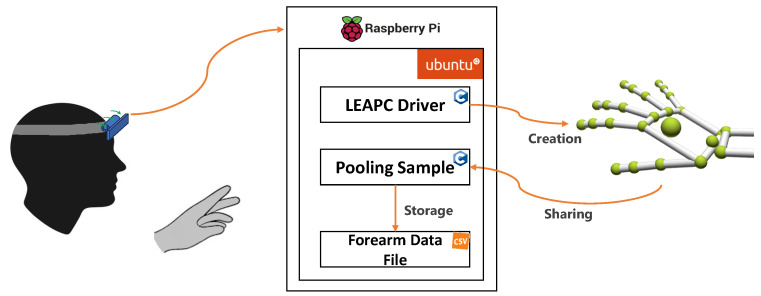
Workflow and interactions between the hardware and software components of the proposed system.

A detailed illustration of how the proposed system operates, including the workflows and the interactions between the hardware and software components, is shown in Figure 5.

The LMC captures hand movements within a three-dimensional coordinate system that is centered around the device itself. The LeapC driver is hosted on the RSP and acquires the raw data from the LMC. From the raw data, the LMC software creates a numerical model of the hand represented as a stick model, reflecting the real anatomy of the human hand, as shown in the right part of Figure 5.

In particular, the LMC data are organized into frames. Each frame represents a distinct snapshot in time and contains all the data captured by the controller at that moment. Within each frame, information about detected hands is provided. This includes data such as hand ID, palm position (x, y, z coordinates), palm velocity, palm normal (the direction the palm is facing), and palm direction (the direction the palm is moving). For each detected hand, detailed information about individual fingers is provided. This includes data such as finger ID, finger type (thumb, index finger, middle finger, ring finger, pinky finger), fingertip position (x, y, z coordinates), finger direction, and finger velocity. Additionally, the LMC can capture raw image data through its infrared cameras. However, these data are primarily used for internal processing and calibration and can potentially be accessed by developers for specialized applications. For this reason, in this work, hand data provided by the API of the LMC are used because they are precise and reliable [18], and the algorithm used by the controller is designed to be efficient for use with applications that require a high frame rate. Finally, the Pooling Sample script establishes the connection with the LeapC driver and receives the LMC data frames. We modified this script to concatenate the data frames, converting each of them into a comma-separated value (CVS) format and saving the data in a table-structured format.

Throughout all the tests conducted, the Graphical User Interface (GUI) was intentionally disabled. This measure was implemented with the dual purpose of conserving energy and minimizing CPU usage. It is crucial to note that this particular characteristic is not a limitation; rather, it aligns with the system’s design intention in scenarios where user–system interaction does not require visual interface elements. For this reason, the absence of a GUI and the option to do away with a monitor altogether serve to optimize the system for its intended purpose.

Figure 4 shows the final embodiment of the proposed system, arranged on a table. As stated above, the monitor is only used for calibration purposes, and for this reason, it is powered by an external power source. In the scenario depicted in Figure 4, the RSP is running the ‘Pooling Sample’ script, which prints the frame number and some specific numerical information regarding the forearms. In these conditions, where the GUI of the OS and the print of the strings in the system terminal are slowing down the system’s performance, the frame rate is about 60 fps, providing near real-time capabilities.

## 4. Measurements

The proposed system is characterized by its portability, for which power consumption estimation is very important because it directly affects battery duration. Additionally, CPU usage and its temperature are other fundamental parameters to consider in portable applications, for which the RSP could be placed in areas with little airflow, such as pockets.

For the above reasons, some measurements, tests, and evaluations are required for the complete characterization of the system. The tests were conducted considering two different scenarios: the RSP without the case and the RSP inside the case with a fan.

The first scenario is necessary to establish baseline measurements to be used for reference. The second scenario is the operative one and represents the real operating conditions.

For each scenario, four different configurations were tested:

1. **RSP is on but without a connected LMC (RSP)**: The RSP is not actively involved in intensive operations or processes, consuming its lower-bound power and generating a lower-bound heat compared to when it is engaged in demanding tasks.
2. **LMC is connected to RSP but not tracking (RSP + LMC)**: Compared to just the RSP, the LeapD daemon is running but data streaming is not requested by any software.
3. **LMC is connected to RSP and data from forearm tracking is requested (RP + LMC + Data)**: Compared to the RSP + LMC task, a script for data acquisition, provided as an example by the LMC manufacturer, is running. The script requests data from the LeapD.
4. **LMC is connected to the RSP, forearm tracking is running, and data are printed on the console (RP + LMC + Data + Print)**: Compared to the RP + LMC + Data task, the script is also printing data on the console. This configuration represents the worst-case (upper-bound) scenario for power consumption since, for the considered portable applications, the monitor is not used.

For all the above configurations, the OS GUI was disabled, leaving only the system terminal rendered on the screen, the latter being powered by an independent power source. The only energy absorbed from the power bank is related to the video data shared through the HDMI port, which we consider negligible.

For these experiments, the proposed system was arranged on a table, and the LMC was placed on the head of the user. For configurations 3 and 4, the user typed on a keyboard and interacted with the smartphone to simulate different hand poses and occlusions. The methodology and results are reported in Section 4.1 and Section 4.2.

Another fundamental aspect to consider is the frame rate of the proposed system. The frame rate of the LMC is variable and dependent on several factors such as CPU availability, lighting conditions, the presence of hand occlusions, etc. For this reason, the proposed system was tested in real operational scenarios, in which the user interacted with different mechanical tools and under different lighting conditions. The methodology and results are reported in Section 4.3.

### 4.1. Power Consumption

For each scenario and configuration, we report the electric voltage, current, and total power consumption. For the measurements, we used a USB tester connected between the USB-C power input of the RSP and the USB-C power output of the power bank. The sensitivities of the USB tester used are 0.01 V, 0.01 A, and 0.01 W.

The data were collected every 10 s for a total of 170 s. Table 1 reports the averaged results for both the baseline scenario (when the RSP was not inside the case and without a fan) and the operating conditions (when the RSP was inside the case and in the presence of a switched-on fan).

Starting from the RSP without the case (baseline scenario), the electric voltage was almost the same across all tasks, ranging between 4.99 V and 5.06 V.

Instead, the absorbed current varied depending on the task. In the first configuration (RSP), the current consumption ranged between 0.50 A and 0.62 A (average 0.53 A), and in the RSP + LMC configuration, it ranged between 0.79 A and 0.95 A (average 0.84 A). Consequentially, based on our measurements, 0.33 A was absorbed by the LMC, confirming the datasheet of the manufacturer, which stated that the LMC can require up to 0.5 A [42]. In fact, in this task, the LeapD was running, and the IR sensors were on, but there was no data request from any application. In the RSP + LMC + Data configuration, the absorbed current ranged between 1.15 A and 1.29 A (average 1.19 A), and finally, in the RSP + LMC + Data + Print configuration, it ranged between 1.26 A and 1.36 A (average 1.30 A). The increased current can mainly be attributed to the increased CPU usage.

Finally, we measured the active power consumption [W], which can be also calculated as P=V∗I, where *V* is the electric voltage measured in volts [V] and *I* is the current measured in amperes [A]. We compared the values of *P* measured with the ones calculated, and the results were the same. When the first configuration was considered (RSP), the average power consumption ranged between 2.55 W and 3.15 W (average 2.68). In the RSP + LMC configuration, it ranged between 3.95 W and 4.75 W (average 4.19 W); in the RSP + LMC + Data configuration, it ranged between 5.75 W and 6.47 W; and in the RSP + LMC + Data + Print configuration, it ranged between 5.75 W and 6.47 W (average 5.97 W). Since, as previously stated, the electric voltage remained constant across all tasks, the differences in power consumption can be attributed to the increment in current consumption, and the same considerations hold.

The same measurements were repeated with the RSP using the operating configuration (fan on). In this case, the electric voltage was almost the same for the RSP and the RSP + LMC configurations, but a slight drop was measured for the RSP + LMC + Data and the RSP + LMC + Data + Print configurations. Regarding current consumption, in the RSP configuration, the values ranged between 0.55 A and 0.65 A (average 0.58 A). Compared with the RSP without a fan, the current consumption increased by 0.05 A. After the connection of the LMC, the average current consumption increased to 1.03 A, ranging between 1.00 and 1.08. Compared with the RSP without a fan, the current consumption increased by 0.19 A. In the RSP + LMC + Data configuration, the average current consumption increased to 1.37 A, ranging between 1.30 and 1.45, with an increase of 0.18 A compared to the same configuration without the fan. Finally, in the RSP + LMC + Data + Print configuration, the average current consumption increased to 1.38 A, ranging between 1.32 and 1.48, with an increase of 0.08 A. On average, compared with the ‘fanless’ configuration, the proposed configuration required 0.13 A, in line with the current consumption stated by the manufacturer of the fan. The increments in power consumption compared to the fanless configuration were 0.23 W, 1.01 W, 0.84 W, and 0.30 W, which equates to an average of 0.60 W. The increased power consumption cannot be totally attributed to the fan. The introduction of the fan has an acceptable impact on power consumption, but as discussed in the next section, it is fundamental to keep the RSP temperature under control.

**Figure 6:**
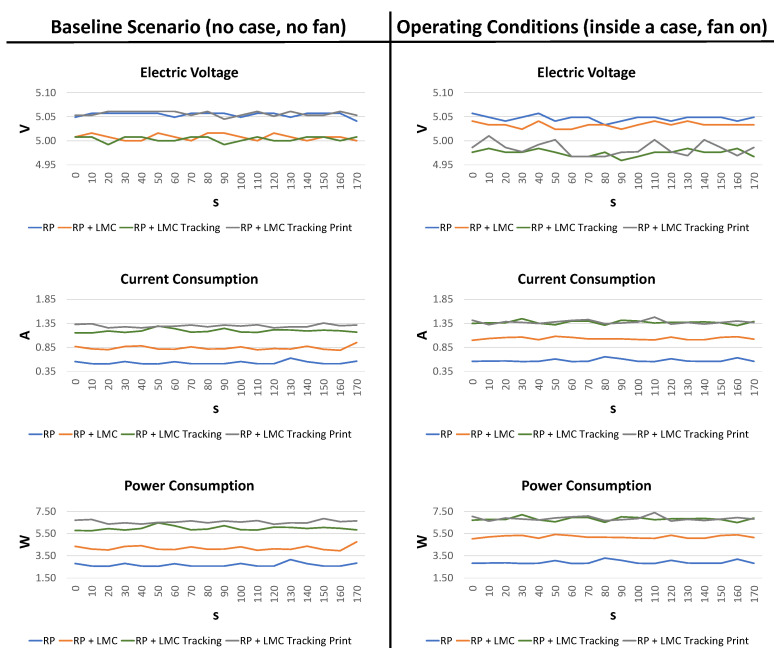
Voltage, current, and power measurements for both considered scenarios.

Finally, Figure 6 reports the plots for the baseline scenario and the operating conditions: the relevant result is that all measurements exhibit stability over time.

### 4.2. CPU Usage and Temperature

For each scenario, we report the CPU usage [%] and the CPU temperature [°C], measured using the RSP’s on-board sensor and the script GetData, as described in Section 3. Compared to the measurements of energy consumption, these measurements required more samples because the temperature took more time to converge to the final value. For this reason, a script was developed to acquire values every 10 s for 890 s. Table 2 reports the results for both the RSP baseline (when the RSP was not inside the case) and the proposed configuration (when the RSP was inside the case with the fan).

**Figure 7:**
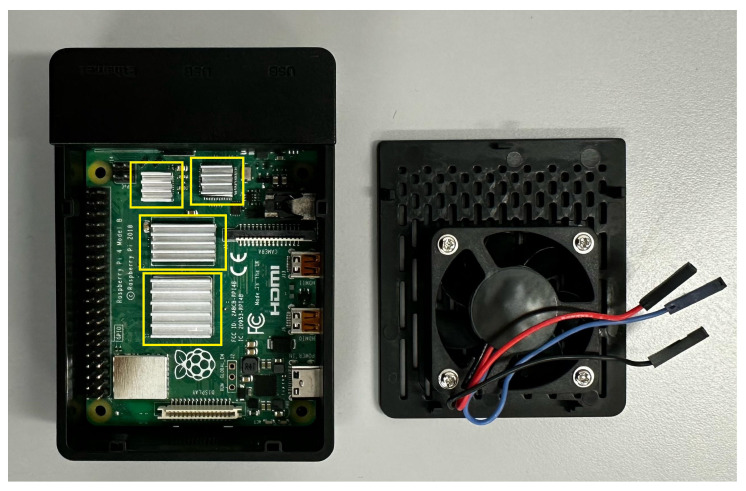
The aluminum heatsinks used during the measurements for the baseline and the proposed assembly are highlighted with yellow boxes. The figure also shows the RSP enclosed in the case and the fan used for cooling in the proposed assembly.

Starting from the baseline scenario, the CPU usage was minimal in the RSP configuration (the GUI was disabled). When the LeapD was running (RSP + LMC), CPU usage was almost 11%, and it reached 74.02% when data were requested from an application (RSP + LMC + Data). When printing was added (RSP + LMC + Data + Print), the average CPU usage increased slightly. Regarding the temperature, in the RSP configuration, it was 53.66 °C and increased for each task: 60.36 °C, 74.75 °C, and 75.49 °C. This demonstrates that without any active cooling, the temperatures reached were too high. In fact, during the most intense task (RSP + LMC + Data + Print), there was a peak temperature of 80.34 °C. As reported in the specifications, the RSP began to throttle the processor when the temperature reached 80 °C, increasing when it reached the maximum temperature of 85 °C [43]. This justifies the adoption of a fan installed on a case. In fact, in the proposed configuration, even though the CPU usage was almost identical to the baseline scenario, the drop in temperature was evident across all tasks. Even in the most intense task, the maximum temperature registered was 58.43 °C, which was almost 22 °C lower than in the same task without the fan, although it is now enclosed in a case. Additionally, in both scenarios, passive adhesive aluminum heatsinks were used, as shown in Figure 7.

**Figure 8:**
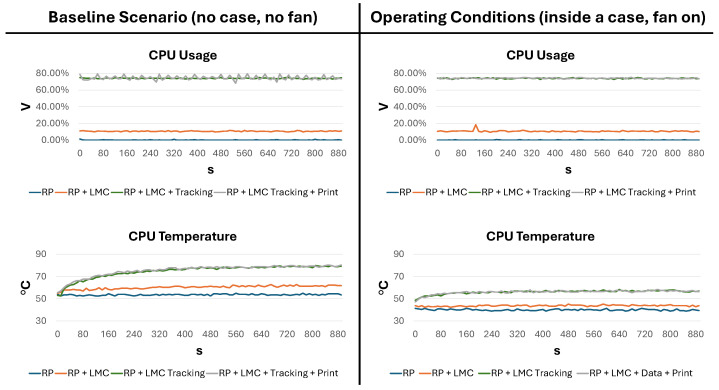
CPU usage (in %) and temperature (in °C) for both scenarios.

Finally, the plots for the baseline scenario and the operating configuration are shown in Figure 8. Although CPU usage was stable, the CPU temperature significantly increased, especially in the baseline scenario. In fact, in the RSP + LMC + Tracking + Print configuration, the temperature exceeded 70 °C after only 160 s and was unstable, showing an increasing trend. In contrast, in the same scenario for the final RSP assembly, after the same amount of time, the temperature had already converged to a stable value at around 58 °C.

The last significant piece of information gathered from using the proposed device in both scenarios was the power bank’s battery duration. We left the system in RSP mode for the baseline scenario until it turned off due to battery expiration, and we found out that the duration was around 12 h. Then, we repeated the same task for the operating conditions (fan on), but now in RSP + LMC + Data + Print mode, the duration was 5 h. The results indicate that for the worst-case scenario, the duration of the battery is completely compatible with most human activities the system is involved in tracking. It is worth noting that for the baseline scenario, we did not perform measurements in the other modalities both because we were interested in the maximum duration of the battery (obtainable when only the RSP is on) and because we did not want to risk damaging the RSP due to heat.

### 4.3. Hand Tracking

In the previous sections, the RSP demonstrated its capability of running an LMC while remaining well within acceptable energy consumption and CPU temperature limits, without saturating the CPU, when an active system is used (fan). As a final test, we acquired data from both forearms in real operative conditions (RSP + LMC + Data) and checked the fps, considering that for real-time, fluid, interactions, the minimal fps value is 25–35 [44,45].

**Figure 9:**
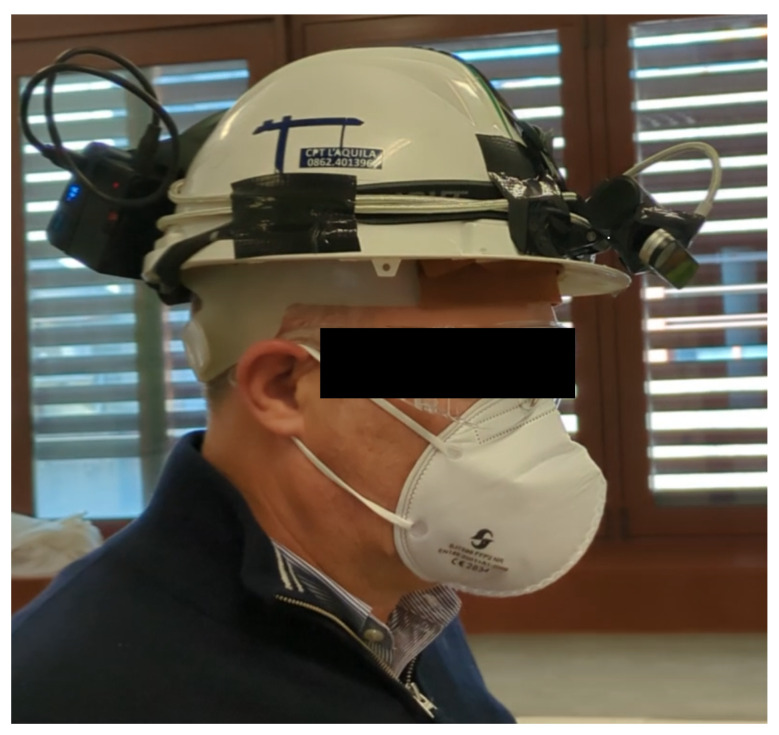
The proposed system mounted on a safety helmet used for testing real-life scenarios.

We used the proposed system in the configuration shown in Figure 9, where the system was mounted on a helmet to improve safety at work (as can be seen, the RSP and the power bank were also fixed on the back part of the helmet). In addition, forearm tracking could be used to check for dangerous forearm positions regarding a mechanic tool and proactively stop the tool in the case of risks.

**Figure 10:**
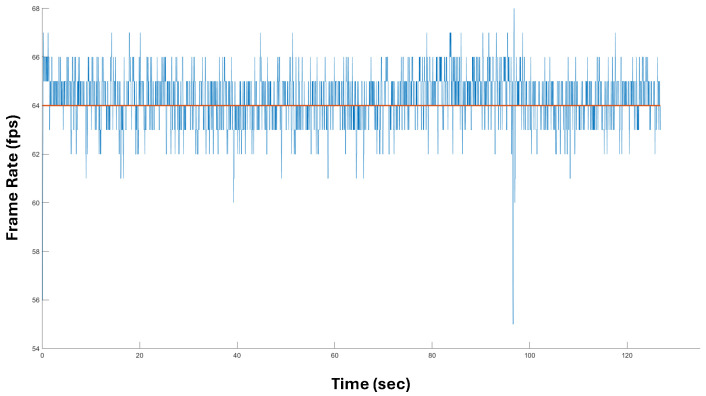
The frame rate of the LMC was read from the internal data structure and reported. The original averaged values also contained decimals, which have been eliminated for convenience.

We tested the proposed system while the forearms were performing different tasks: simple movements in the air (the person was standing up), typing on a keyboard (the person was sitting), and interacting with a manual tool (the person was walking around the room while performing tool tapping). For the latter task, the illumination conditions varied due to the fact the person was moving near a window, then in the center of the room, and finally, near the illumination of a lamp. This is an important aspect because the LMC is based on the use of infrared light to operate: external sources of infrared light could negatively affect tracking precision. Furthermore, in this situation, the software activates specific countermeasures to compensate for this, requiring considerable CPU overload [46]. However, in our test, the results of which are reported in Figure 10, the proposed system was capable of operating at an average of 64 fps, with values ranging between 62 fps and 66 fps. Only in one isolated case did it drop to 55 fps, corresponding to a fast passage close to the window. Ultimately, the system always operated above 30 fps, thus ensuring real-time tracking.

**Figure 11:**
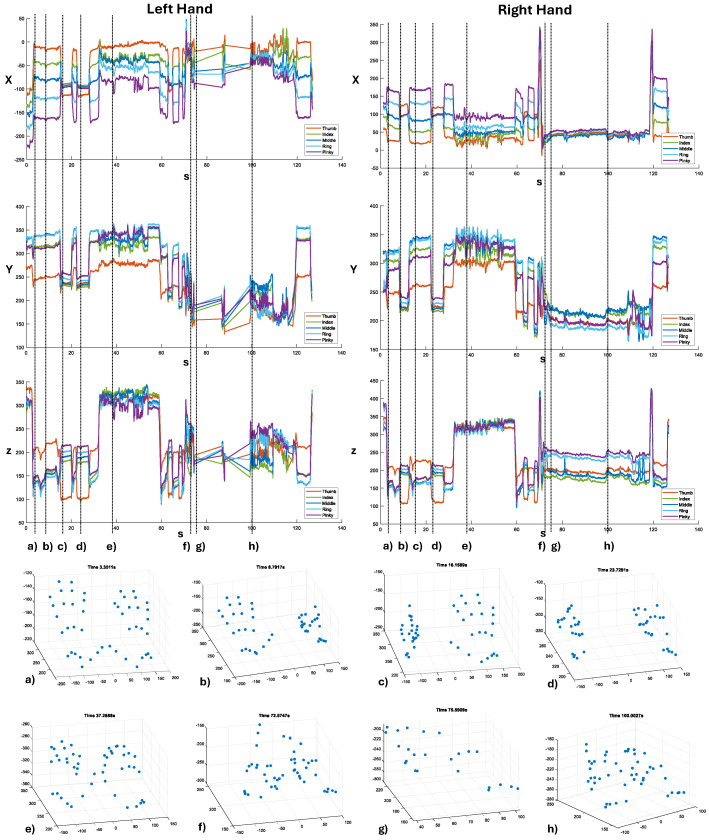
The plots of the spatial positions of each fingertip of both hands as measured by the LMC. To improve visualization, measurements for each spatial coordinate are shown in separate plots. For each coordinate, measurements of the fingertips from the same hand are shown in the same plot with different colors to facilitate comparison. The vertical dotted lines represent particular hand poses (shown in the scatter plots from (**a**–**h**) to better contextualize the plots.

Figure 11 shows the spatial coordinates (X, Y, Z) of the fingertips of both hands throughout the data acquisition, corresponding to the movements analyzed for fps in Figure 10. To enhance the understanding of the plots, vertical lines are drawn corresponding to specific hand poses, as shown in plots (a) to (h). The plots were created using Matlab 2023a, with blue dots used to mark the joints of the hands acquired by the LMC. For the reported poses, movements were smooth except when the person was interacting with the manual tool (Figure 11f). In this case, the left hand was almost completely obscured by the tool, and the LMC lost track at some point (Figure 11g). Regarding the right hand, tracking was more stable because most of the time, the person was interacting with the index finger, and consequently, the hand was not hidden behind the tool, making it visible to the LMC. However, self-occlusions also occurred, where some fingers obstructed the view of others. In these cases, hand-tracking precision decreased significantly, and jumps occurred when the LMC recovered the right tip position.

## 5. Conclusions and Future Work

In this work, we proposed a portable hand-tracking system based on a head-mounted LMC connected to an RSP. The system is powered by a 10,000 mAh power bank. Several measurements were conducted. Regarding power consumption, the system is capable of running for 5 h in tracking mode. CPU usage is not saturated, leaving space to run other applications. The temperature is very well controlled using heatsinks and a fan, even under the most intense load, which, in this case, occurs when the LMC is tracking and external software is requesting the frames and saving the data. Regarding frame rate, the system is capable of running at an average of about 60 fps without any significant drops. Tracking precision is good and stable, as demonstrated by the test we conducted under changing operative conditions, although overall precision could be affected.

Considering these results, it can be confidently asserted that the proposed system holds significant promise for various precise hand-tracking applications. Head-mounted hand tracking emerges as a particularly effective design choice due to its inherent advantages: users can freely engage with their surroundings without being hindered by the position of the hand-tracking sensor, which consistently aligns with their field of view. This freedom allows users to grasp real-world objects, fostering a natural and immersive interaction experience. Additionally, the system’s portability has been substantiated by its ability to seamlessly integrate the computing power of the RSP with the demands of the LMC, alongside a battery life that accommodates all major tasks.

Future work will be in the direction of definitive system engineering. First of all, a button to start and stop hand tracking will be added. This will enable the possibility of pausing tracking when it is not necessary, allowing significant energy savings and, consequently, increasing usage time.

To further increase the efficiency of the system, the velocity of the fan will be controlled, taking into account the temperature of the CPU. To minimize fan usage, heatsinks with different shapes and materials with better thermal conductivity will be tested.

Another aspect is to implement data streaming using Wi-Fi. In this way, it will be possible to run intensive computations like deep learning on a dedicated workstation and retrieve the results, significantly extending the range of potential applications [47]. After these improvements, the system will be extensively tested in real-world applications involving gesture recognition and action prediction in both indoor and outdoor scenarios.

Hand-tracking technology holds promise across various practical applications, particularly in healthcare. It can enhance hand rehabilitation by providing detailed quantitative analysis and feedback on hand movements [1,2,3,47]. Additionally, it can contribute to research endeavors focused on motor imagery development when hand motor execution data could be useful, especially when integrated with tools like electroencephalography [48]. Beyond healthcare, hand tracking has implications in gaming [49], human–computer interaction [50], and security/authentication systems [51].

## Figures and Tables

**Table 1:** Voltage, current, and power consumption in two different scenarios: Baseline scenario (left) and operating conditions (right). In the operating conditions, the use of a fan is also considered to maintain a low operating temperature. Averaged measurements are reported.

	Baseline Scenario (No Case, No Fan)				Operating Conditions (Inside a Case, Fan on)			
	RSP	RSP + LMC	RSP + LMC + Data	RSP + LMC + Data + Print	RSP	RSP + LMC	RSP + LMC + Data	RSP + LMC + Data + Print
**V**	Avg: 5.05 Min: 5.04 Max: 5.06	Avg: 5.01 Min: 5.00 Max: 5.02	Avg: 5.00 Min: 4.99 Max: 5.01	Avg: 5.06 Min: 5.05 Max: 5.06	Avg: 5.05 Min: 5.03 Max: 5.06	Avg: 5.03 Min: 5.02 Max: 5.04	Avg: 4.97 Min: 4.96 Max: 4.98	Avg: 4.98 Min: 4.97 Max: 5.01
**A**	Avg: 0.53 Min: 0.50 Max: 0.62	Avg: 0.84 Min: 0.79 Max: 0.95	Avg: 1.19 Min: 1.15 Max: 1.29	Avg: 1.30 Min: 1.26 Max: 1.36	Avg: 0.58 Min: 0.55 Max: 0.65	Avg: 1.03 Min: 1.00 Max: 1.08	Avg: 1.37 Min: 1.30 Max: 1.45	Avg: 1.38 Min: 1.32 Max: 1.48
**W**	Avg: 2.68 Min: 2.55 Max: 3.15	Avg: 4.19 Min: 3.95 Max: 4.75	Avg: 5.97 Min: 5.75 Max: 6.47	Avg: 6.55 Min: 6.34 Max: 6.85	Avg: 2.91 Min: 2.80 Max: 3.29	Avg: 5.20 Min: 5.02 Max: 5.42	Avg: 6.81 Min: 6.49 Max: 7.20	Avg: 6.85 Min: 6.62 Max: 7.39

**Table 2:** CPU usage (in %) and temperature (in °C) for both considered scenarios.

	Baseline Scenario				Operating Conditions (Fan on)			
	RSP	RSP + LMC	RSP + LMC + Data	RSP + LMC + Data + Print	RSP	RSP + LMC	RSP + LMC + Data	RSP + LMC + Data + Print
**%**	Avg: 0.14 Min: 0.00 Max: 1.30	Avg: 10.63 Min: 9.80 Max: 11.60	Avg: 74.02 Min: 72.80 Max: 75.10	Avg: 74.57 Min: 68.10 Max: 79.50	Avg: 0.10 Min: 0.00 Max: 0.70	Avg: 10,67 Min: 9.30 Max: 18.40	Avg: 73.95 Min: 72.70 Max: 74.90	Avg: 74.09 Min: 73.00 Max: 75.20
**°C**	Avg: 53.66 Min: 52.58 Max: 55.02	Avg: 60.36 Min: 56.97 Max: 62.81	Avg: 74.75 Min: 52.58 Max: 79.85	Avg: 75.49 Min: 55.50 Max: 80.34	Avg: 40.05 Min: 38.95 Max: 41.38	Avg: 43.77 Min: 42.35 Max: 45.28	Avg: 56.06 Min: 48.69 Max: 58.27	Avg: 56.04 Min: 47.23 Max: 58.43

## Data Availability

Data are contained within the article.
